# Transplantation of endothelial progenitor cells attenuated paraquat-induced acute lung injury via miR-141-3p-Notch-Nrf2 axis

**DOI:** 10.1186/s13578-018-0219-1

**Published:** 2018-03-20

**Authors:** Yan Jin, Wei Liu, Xiaowei Liu, Tao Ma, Chen Yang, Quan Cai, Zhi Liu

**Affiliations:** Department of Emergency, The First Affiliated Hospital of China Medical University, No. 155 Nanjing North Street, Heping District, Shenyang, 110001 People’s Republic of China

**Keywords:** EPC, miR-141-3p, Notch-Nrf2 axis, PQ, ALI

## Abstract

**Background:**

Paraquat (PQ) presents with high toxicity for humans and animals, and the lungs become the main target organ by the poisoning of PQ leading to acute lung injury. Endothelial progenitor cells (EPCs) were proved to have the repair function on acute lung injury (ALI). We aimed to invatigate the underlying mechanism of EPCs in PQ-induced ALI involving miR-141-3p.

**Methods:**

Endothelial progenitor cells were isolated from peripheral blood of C57BL/6J mice and identified by flow cytometry. Lung wet-to-dry (W/D) weight ratios, lung injury score and the number of total leukocyte and the number of neutrophils in BALF were used to analyze the degree of lung injury. The transfection was performed with Lipofectamine 2000. The levels of miRNA and mRNA were determined by qRT-PCR, and the protein levels were detected by Western blot assay.

**Results:**

Endothelial progenitor cells alleviated lung wet-to-dry (W/D) weight ratios, lung injury score and the number of total leukocyte and the number of neutrophils in BALF in PQ-induced ALI mice. EPCs inhibited miR-141-3p expression, and enhanced the levels of Notch-Nrf2 axis in PQ-induced ALI mice. MiR-141-3p knockdown reversed the PQ induced-inhibition on Notch-1 and Hesr1 expression. MiR-141-3p over-expression could inhibit the expression of Notch-1 pathway significantly in the pulmonary epithelial cell line MLE-12. Both miR-141-3p over-expression and si-Notch-1 abolished the protection effect of EPCs on lung injury induced by PQ in vivo.

**Conclusions:**

Endothelial progenitor cells could provide therapeutic effect on PQ-induced ALI via miR-141-3p-Notch-Nrf2 Axis.

## Background

As the world-wide and nonselective contact quaternary nitrogen herbicide [1], paraquat (1,1-dimethyl-4,4-bipyridilium dichloride, PQ) presents with high toxicity for humans and animals [2, 3]. PQ is selectively accumulated in the lungs after ingested, so the lungs become the main target organ by the poisoning of PQ leading to acute lung injury (ALI) hallmarked by a edema, hemorrhage, interstitial inflammation, interstitial inflammation, and progressive fibrosis [4, 5]. Lung injury causing respiratory failure is the major reason of PQ-induced death. The inflammatory reaction, a type of redox reactions, which induced disruption of cellular membranes leading to PQ toxic effects [6], is the main physiological hallmark of PQ-induced ALI [7]. However, there is no specific antidote for the treatment for PQ-induced ALI.

Endothelial progenitor cells (EPCs) are the endothelial cell precursors derived from bone marrow, with the capacity to differentiate into endothelial cells and take part in vasculogenesis in the sites of neovascularization [8]. With the in-depth research, EPCs were reported to play a vital role in vasculogenesis and the maintenance of vascular integrity and show the potential to protect the structure of lung in smokers [9, 10]. In addition, EPCs were proved to have the repair function of restoring denuded parts of the injured artery and directly emerging new vessels to sustain the completeness of the endothelial monolayer in animal model [11]. Meanwhile, in the rabbit endotoxin-induced ALI models, EPCs have the effects in protecting the endothelial function and sustaining the integrity of alveolar-capillary barrier of lungs [12]. Hence, we hypothesized that EPCs might play a role in PQ-induced ALI.

MicroRNA (miRNA) with the length of 20–22 NT, one type of non-coding RNA, have the function of negatively regulating the genes at post-transcriptional level to participate in many biological process [13]. It is no exception that miR-141-3p plays the important roles in many physiological processes and its deregulation was involved in many diseases. As reported by Shen et al. [14], miR-141-3p could relieve chronic inflammatory pain via down-regulation of downstream target gene HMGB1. IGF-2 was regulated by miR-141-3p to influence the activation of Akt in the mouse placental development [15]. In addition, it was reported that Notch-1 were the downstream target gene of miR-141-3p in the Ovaries [16]. In this study, we aimed to invatigate the underlying mechanism of EPCs in PQ-induced ALI involving miR-141-3p.

## Methods

### Isolation, characterization and delivery of EPCs

3 mL/kg peripheral blood was obtained from the C57BL/6J mice. Mononuclear cells (MNCs) were isolated by Ficoll-Plaque Plus (Amersham Biosciences) with density gradient centrifugation. MNCs were then rinsed and seeded on 6-well plates coated with human fibronectin (Sigma-Aldrich) and supplemented with EGM-2 (Cambrex). On 7 days of culture, the adherent cells (as early EPCs) were collected for transplantation. The early EPCs were maintained subsequently with acetyl-LDL (10 µg/mL; Molecular Probes, Carlsbad, USA) and isolectin (5 µg/mL; Molecular Probes). The staining of acetyl-LDL and isolectin in EPCs was determined under fluorescence confocal microscopy with the absorption wave lengths of 555 and 495 nm, respectively. EPCs (3 × 10^5^ cells) were harvested for trypsinization 7 days after seeding for transplantation.

### Flow cytometry

For identifying CD31 + CD34 + Flk-1 + CD45 − CD133-cells, the EPCs were maintained with combinations of FITC-conjugated mouse anti-human CD31, CD34 and CD133, or an APC-conjugated anti-human CD45 and Flk-1 for 30 min at 4 °C; subsequently, these cells were rinsed twice with PBS. The immunoglobulin G isotype antibodies were used as negative controls. Then, the number of CD31 + CD34 + Flk-1 + CD45 − CD133-cells was detected with FACSort flow cytometer (Beckman Biosciences). All the staining was carried out in accordance with manufacturer’s protocols.

### Experimental animals

C57BL/6J mice (gender: male, weight: 25–30 g, age: 10–12 weeks) were provided by the Animal Center of China Medical University for the experiments. In addition, experiments on animals obtained the approval of the experimental animal ethical committee of China Medical University. For acclimation for the lab condition, all mice were raised with pathogen-free facility and 12 h day and night cycle at 23–25 °C and were with access to free food and water.

### Experimental protocols

After subjected to acclimatization of a week, all mice were equally divided into seven groups: the control group (mice were received PBS injection by through tail vein, n = 10), the 10 × 10^6^ EPCs group (mice were injected with approximately 10 × 10^6^ EPCs in 50 μL PBS through tail vein, n = 10), the PQ group (mice were received the intraperitoneal injection of PQ 50 mg/kg, n = 10), the PQ + 1.25 × 10^6^ EPCs group (mice were received the injection of PQ, and then received EPCs injection after 6 h, n = 10), the PQ + 2.5 × 10^6^ EPCs group (mice were received the injection of PQ, and then received EPCs injection after 6 h, n = 10), the PQ + 5 × 10^6^ EPCs group (mice were received the injection of PQ, and then received EPCs injection after 6 h, n = 10), the PQ + 10 × 10^6^ EPCs group (mice were received the injection of PQ, and then received EPCs injection after 6 h, n = 10).

In the following experiments, the mice were randomly distributed into four groups: the PQ group (n = 10), the PQ + EPCs group (n = 10), the EPCs group (n = 10), the PQ + EPCs + pre-NC group (mice were received the injection of PQ, and then received EPCs injection and pre-NC after 6 h, n = 10), the PQ + EPCs + miR-141-3p mimic group (mice were received the injection of PQ, and then received EPCs injection and miR-141-3p mimic after 6 h, n = 10).

In the following experiments, the mice were randomly distributed into four groups: the PQ group (n = 10), the PQ + EPCs group (n = 10), the EPCs group (n = 10), the PQ + EPCs + si-control group (mice were received the injection of PQ, and then received EPCs injection and si-control after 6 h, n = 10), the PQ + EPCs + si-Notch-1 group (mice were received the injection of PQ, and then received EPCs injection and si-Notch-1 after 6 h, n = 10).

### Lung wet-to-dry (W/D) weight ratios

The whole lungs were removed following the mice killed. Each lung was dried, weighed, and then placed in an oven at 80 °C for 48 h to acquisition the “dry” weight. The ratio of the weight of the wet lung to the dry one of the same lung was calculated to evaluate tissue edema [17].

### Lung histopathology

Mice lungs were removed and stored in 4% paraformaldehyde at 4 °C for 48 h. Hematoxylin and eosin staining was performed in accordance with the regular staining method, and the slides were assessed with a semi-quantitative scoring method. Lung injury was scalar in a blinded fashion from 0 to 3 (0, absent; 1, mild; 2, moderate; and 3, prominent) for airway epithelial necrosis, intra-alveolar edema, hyaline membranes, hemorrhage, and the recruitment of inflammatory cells to the air space. The lung injury score was calculated via that the individual scores of each category were added up [17].

### Measurement of leukocytes and proportion of PMNs in BALF

Bronchoalveolar lavage fluid (BALF) samples from each group of mice were simultaneously collected. A total of 0.5 mL BALF was collected and total cells and neutrophils were counted using a hemocytometer in a double-blind manner for the measurement of leukocytes and the proportion of polymorphonuclear neutrophils.

### Cell culture and transfection

MLE-12 cells, the mice pulmonary epithelial cell line, were purchased from Shanghai Institute of Cell Biology (Shanghai, China) and were maintain in DMEM (Hyclone, UT, USA) containing 10% FBS (Gibco, MD, USA). To establish in vitro model, MLE-12 cells were suffered from the exposure of 100 μM PQ for 24 h and the control cells were maintained in normal DMEM. To assess the effect of miR-141-3p/Notch axis on mice pulmonary epithelial cells, si-Notch-1/si-control, miR-141-3p inhibitor/miR-NC (negative control) synthesized by RiboBio (Guangzhou, China) and Lipofectamine 2000 (Invitrogen, Carlsbad, USA) were attenuated with Opti-MEM, respectively. Then the attenuated genes and Lipofectamine 2000 were mixed, followed by used to transfect the MLE-12 cells or the PQ-treated MLE-12 cells.

### The determination of miRNA and mRNA

Trizol (Invitrogen, United States) were used to extract and purify the total RNA. To evaluate the level of miRNAs, the SYBR *Premix Ex Taq*II of SYBR PrimeScript™ miRNA RT-PCR kit (Takara, Dalian, China) was used to reverse transcribed synthesis of cDNA. The remaining steps of qRT-PCR were used with SYBR PrimeScript™ miRNA RT-PCR kit. To assess the mRNA levels of Notch-Nrf2 pathway, the MMLV Reverse Transcriptase First Strand cDNA Synthesis Kit (Invitrogen, Carlsbad, USA) was used to synthesize cDNA. The real-time PCR was performed with SYBR Green Real-time PCR kit (Takara, Dalian, China) on Applied Biosystems 7900HT Sequence Detection System (United States). U6 acted as internal control for normalization of miRNAs and GAPDH were selected as the internal control for normalization of Notch-Nrf2 pathway. The relative levels of gene expression were analyzed by 2^−ΔΔCt^ method [18].

### Western blot assay

The mice lung tissues and MLE-12 cells were performed to Western blot to examine the levels of Notch-Nrf2 pathway in accordance with standard protocols [19]. Membranes were incubated with appropriate primary antibodies against Notch-1, Hesr1, Nrf2, Hmox1, and Txnrd1 (Abcam, USA) and secondary antibodies (Santa Cruz, USA). β-Actin (Sigma, USA) was chosen as internal control. Millipore’s enhanced chemiluminescence kit was used for the visualization of proteins.

### Statistical analysis

All data were statistically analyzed with Student-t test (comparing two groups) and one way analysis of variance (ANOVA; comparing more than two groups) on the GraphPad Prism 5 software (San Diego, CA, USA) and were exhibited as mean ± SD. Values of P less than 0.01 were considered significant.

## Results

### EPCs alleviated the lung injury induced by paraquat (PQ) in vivo

**Fig. 1:**
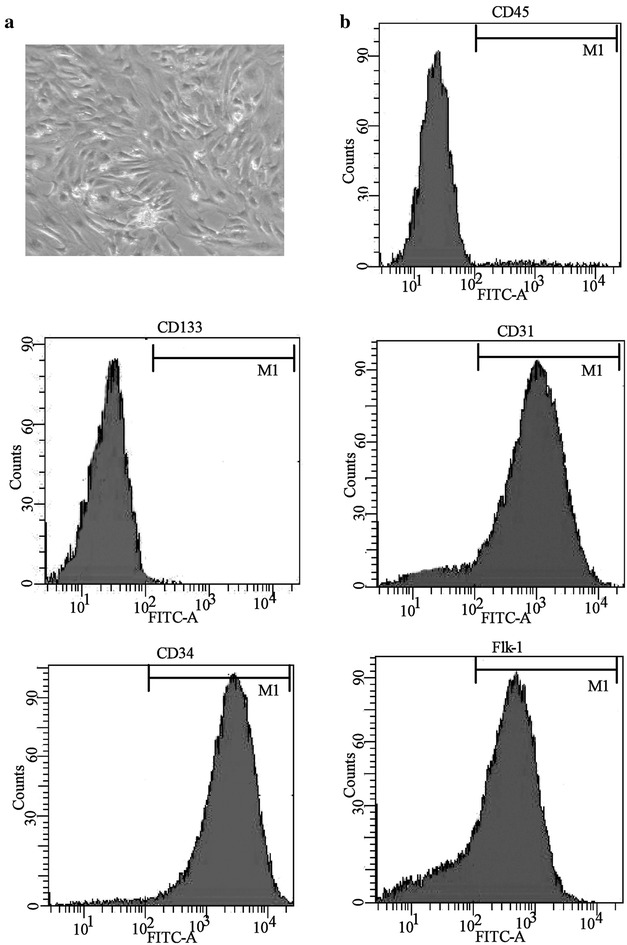
Characterization of endothelial progenitor cells (EPCs). **a** The morphology of EPCs on day 7 of the culture. **b** EPCs were identified by flow cytometry

**Fig. 2:**
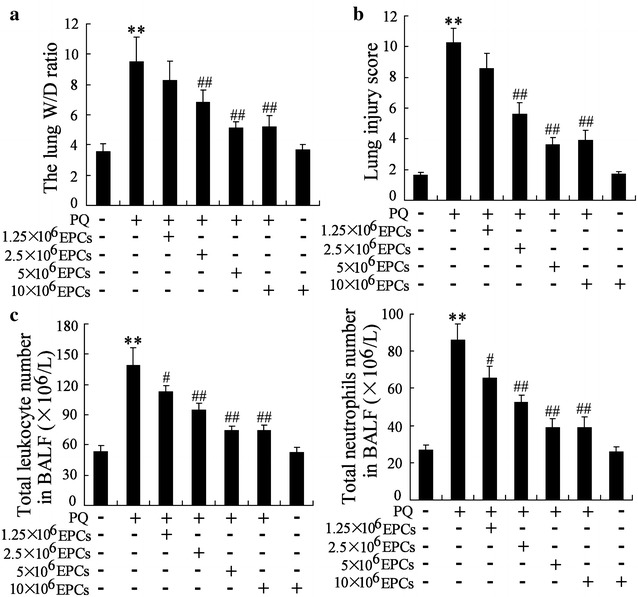
EPCs alleviated the lung injury induced by paraquat (PQ) in vivo. C57BL/6J mice were randomly divided into four groups (n = 10 per group). The control group, mice received PBS by through tail vein injection; the EPCs group, mice were injected with EPCs (approximately 5 × 10^6^ in 50 μL PBS through tail vein injection); the 10 × 10^6^ EPCs group, mice were injected with approximately 10 × 10^6^ EPCs in 50 μL PBS through tail vein; the PQ group, mice were received the intraperitoneal injection of PQ 50 mg/kg; the PQ + 1.25 × 10^6^ EPCs group, mice were received the injection of PQ, and then received EPCs injection after 6 h; the PQ + 2.5 × 10^6^ EPCs group, mice were received the injection of PQ, and then received EPCs injection after 6 h; the PQ + 5 × 10^6^ EPCs group, mice were received the injection of PQ, and then received EPCs injection after 6 h; the PQ + 10 × 10^6^ EPCs group, mice were received the injection of PQ, and then received EPCs injection after 6 h. **a** The lung W/D ratio, **b** lung injury score and **c** the number of total leukocyte and the number of neutrophils in BALF of the mice were detected. **P < 0.01 vs. control; ^##^P < 0.01 vs. PQ

In the present study, endothelial progenitor cells (EPCs) had been successfully isolated and cultured in vitro, and EPCs were confirmed by morphological observation and the surface molecules with flow cytometry. According to Fig. 1a, b PQ (50 mg/kg i.p.) increased significantly the lung W/D ratio, lung injury score and the number of total leukocyte and the number of neutrophils in BALF of mice, while the lung injury induced by PQ were eased obviously by the injection of EPCs, suggesting that EPCs could alleviate the lung injury induced by PQ in vivo. Moreover, the protective effect by EPC was gradually increased with the increasing cell number, and 5 × 10^6^ EPCs exhibit the best protective effect on injured lung (Fig. 2).

### EPCs abolished the effect of PQ on the expression of miR-141-3p, Notch-1 and Nrf2

**Fig. 3:**
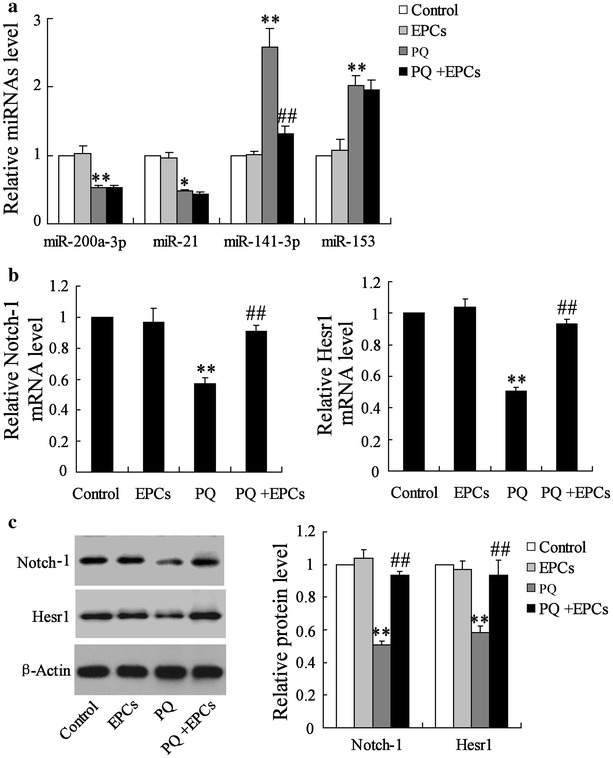
The expression profile of miRNAs in damaged lung tissues induced by PQ. **a** The expressions of miR-200a-3p, miR-21, miR-141-3p and miR-153 in lung tissues were determined using qRT-PCR. **b**, **c** The expressions of Notch-1 and Hesr1 in lung tissues were detected at both the mRNA and protein levels. **P < 0.01 vs. control; ^##^P < 0.01 vs. PQ

**Fig. 4:**
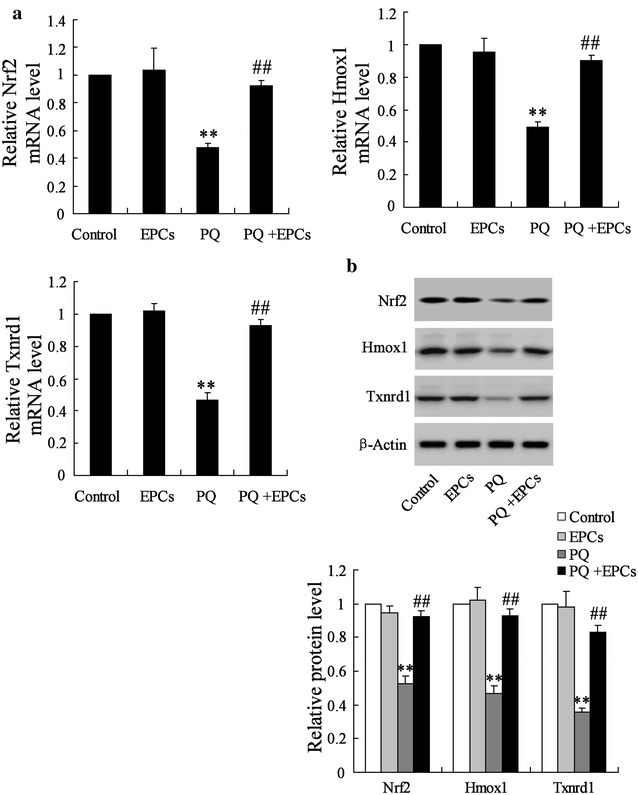
The expression profile of miRNAs in damaged lung tissues induced by PQ. **a**, **b** The expressions of Nrf2, Hmox1 and Txnrd1 in lung tissues were detected at both the mRNA and protein levels. **P < 0.01 vs. control; ^##^P < 0.01 vs. PQ

The expression profile of miRNAs, Notch-1 and Nrf2 in the damaged lung tissues induced by PQ were detected. The results revealed that PQ could inhibit the expression of miR-200a-3p and miR-21, but up-regulated the levels of miR-141-3p and miR-153 in damaged lung tissues; EPCs injection significantly attenuated the PQ induced-up-regulation of miR-141-3p (approximate to 60%), while EPCs had no significant effect on the expression of miR-200a-3p, miR-21 and miR-153 (Fig. 3a). Moreover, PQ down-regulated the expression of Notch-1 and Hesr1 in lung tissues at both the mRNA (respectively to 57 and 51%) and protein levels (Fig. 3b, c), as well as having an inhibitory effect on Nrf2, Hmox1 and Txnrd1 (respectively to 47, 49 and 46% at mRNA levels) expression (Fig. 4a, b). However, EPCs reversed the PQ induced-inhibition on Notch-1, Hesr1, Nrf2, Hmox1 and Txnrd1 expression (Figs. 3b, c; 4a, b).

### PQ regulated Notch-Nrf2 axis through miR-141-3p in vitro

**Fig. 5:**
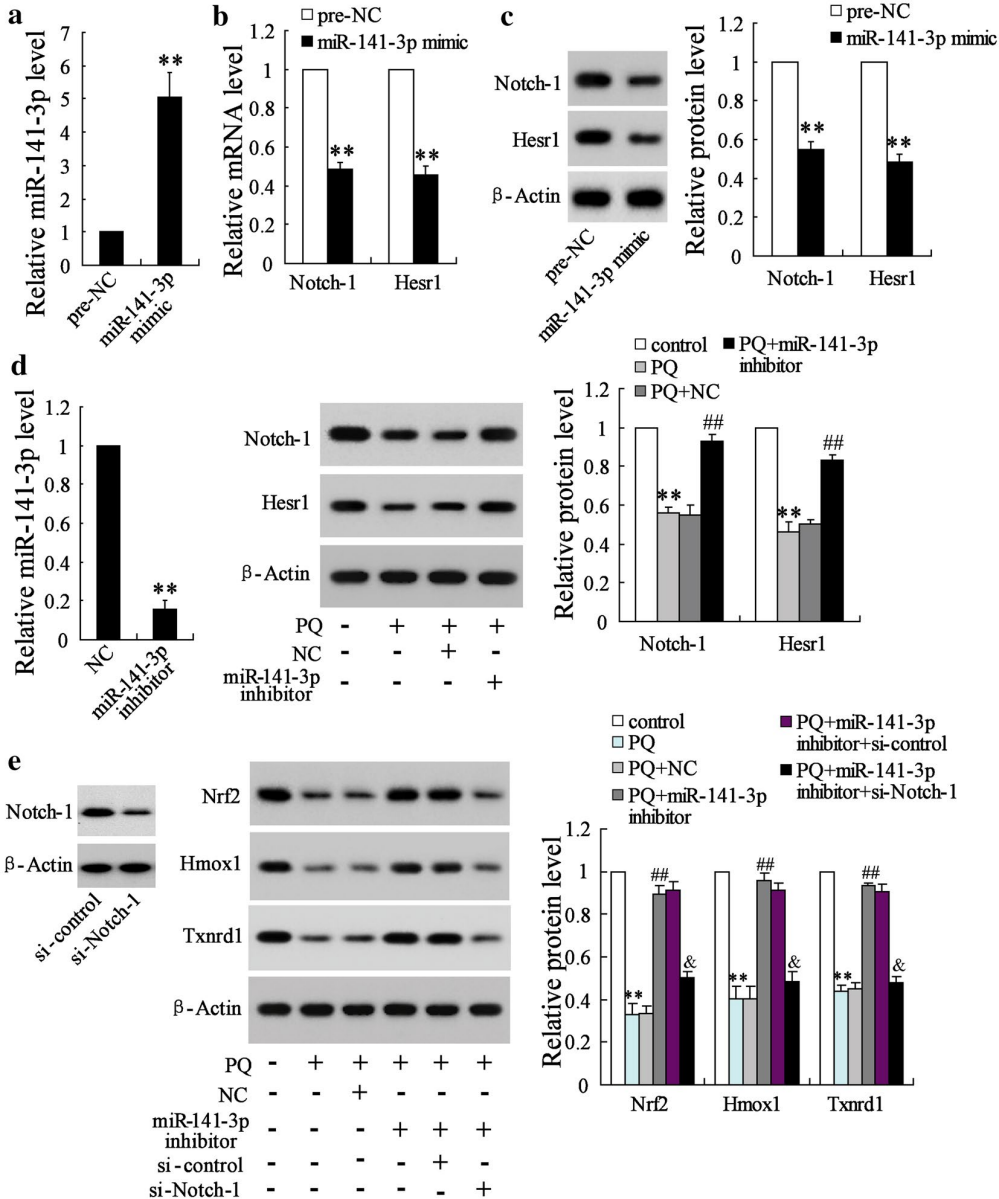
The interaction between miR-141-3p and Notch-1 in pulmonary epithelial cells. **a** The pulmonary epithelial cell line MLE-12 was transfected with miR-141-3p mimic or its control (pre-NC), and **b** the mRNA and **c** protein expressions of Notch-1 and Hesr1 were then determined. **d** MLE-12 cells were transfected with miR-141-3p inhibitor or its control (NC) and then treated by PQ. The expressions of Notch-1 and Hesr1 protein were detected using western blot. **e** MLE-12 cells were divided into six groups, which were received diverse treatment: MLE-12 cells cultured in normal condition; MLE-12 cells treated by PQ; MLE-12 cells transfected with miR-141-3p inhibitor or its control (NC) and then treated by PQ. MLE-12 cells co-transfected with miR-141-3p inhibitor and si-Notch-1 or si-control and then treated by PQ. The expressions of Nrf2, Hmox1 and Txnrd1 protein were detected using western blot. **P < 0.01 vs. pre-NC

MiR-141-3p over-expression via the transfection of miR-141-3p mimic (Fig. 5a) could inhibit the expression of Notch-1 (to 49% at mRNA level and 55% at protein level) and Hesr1 (to 46% at mRNA level and 48% at protein level) significantly in the pulmonary epithelial cell line MLE-12 (Fig. 5b, c). However, miR-141-3p knockdown via the transfection of miR-141-3p inhibitor reversed the PQ induced-inhibition on Notch-1and Hesr1 expression (Fig. 5d). Moreover, miR-141-3p knockdown also reversed the PQ induced-inhibition on Nrf2, Hmox1 and Txnrd1 expression, while si-Notch-1 further considerably reduced the effect of miR-141-3p knockdown on Nrf2, Hmox1 and Txnrd1 expression (Fig. 5e).

### The protection effect of EPCs on the PQ induced lung injury was mediated by miR-141-3p-Notch-Nrf2 axis in vivo

**Fig. 6:**
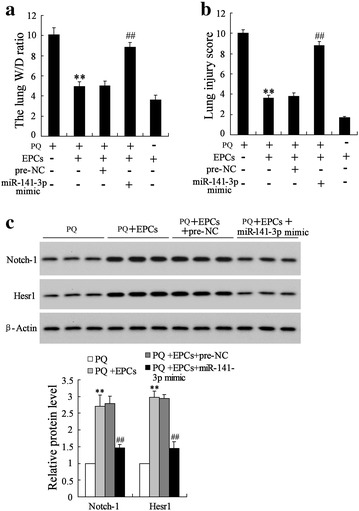
MiR-141-3p over-expression abolished the protection effect of EPCs on lung injury induced by PQ in vivo. C57BL/6J mice were randomly divided into four groups (n = 10 per group), the PQ group; the PQ + EPCs group; the EPCs group; the PQ + EPCs + pre-NC group; the PQ + EPCs + miR-141-3p mimic group. **a** The lung W/D ratio and **b** lung injury score of the mice were detected. **c** The expressions of Notch-1 and Hesr1 protein in lung tissues were detected using western blot. **P < 0.01 vs. PQ; ^##^P < 0.01 vs. PQ + EPCs + pre-NC

**Fig. 7:**
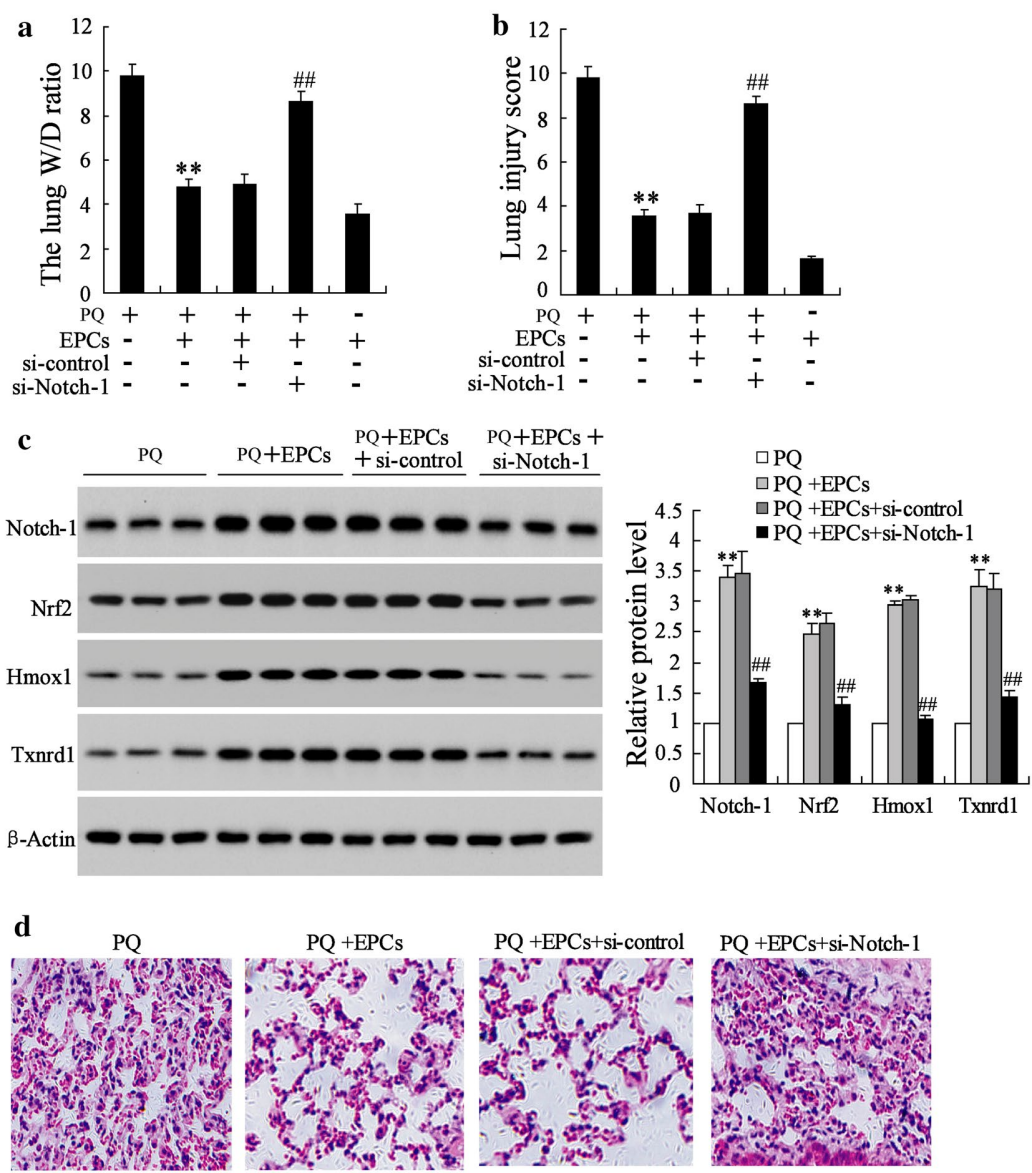
Notch-1 silencing abolished the protection effect of EPCs on lung injury induced by PQ in vivo. C57BL/6J mice were randomly divided into four groups (n = 10 per group), the PQ group; the PQ + EPCs group; the EPCs group; the PQ + EPCs + si-control group; the PQ + EPCs + si-Notch-1 group. **a** The lung W/D ratio and **b** lung injury score of the mice were detected. **c** The expressions of Notch-1, Nrf2, Hmox1 and Txnrd1 protein in lung tissues were detected using western blot. The representing section images of the injured lung tissues are shown in (**d**). **P < 0.01 vs. PQ; ^##^P < 0.01 vs. PQ + EPCs + si-control

According to lung function tests including the lung W/D ratio and Lung injury score, both miR-141-3p over-expression and si-Notch-1 abolished the protection effect of EPCs on lung injury induced by PQ in vivo (Figs. 6a, b; 7a, b, d). MiR-141-3p over-expression also significantly decreased the protein expression of Notch-1and Hesr1 in lung tissues (Fig. 6c). Furthermore, si-Notch-1 significantly attenuated the EPCs induced-up-regulation of Notch-1 protein, as well as Nrf2, Hmox1 and Txnrd1 expression in damaged lung tissues (Fig. 7c).

## Discussion

The toxicity of PQ attributes to the induced redox cycling resulting to oxidative stress-induced inflammation and death of cell [15]. Due to the easy accumulation of PQ in the lung, it induced pulmonary edema, hemorrhage and interstitial inflammation eventually leading to the depression of lung function [4]. The same outcome in our study is that PQ induced pulmonary edema and the severe injury of lung function in mice. EPCs were demonstrated to enhance the rabbit lung function via improving the degree of pulmonary edema and gas exchange, and remit the inflammatory responses in the ALI rabbit lung [20]. Moreover, EPCs markly elevated the function of lung allograft and enhanced survival in the porcine lung transplantation model with prolonged ischemia [11]. In our study, EPCs relieved the PQ-induced ALI via improving the degree of pulmonary edema.

Dysregulated expression of miRNA was commonly involved in various acute injuries of organs, including acute lung injury [21–23]. MiR-1246 modulated pulmonary endothelial cell apoptosis through targeted regulating angiotensin-converting enzyme 2 (ACE2) in LPS-induced ALI [24]. MiR-34a was up-regulated and inhibited the excessive autophagic activity in AT-II cells via targeting FoxO3 to attenuate LPS-induced ALI [25]. Down-regulation of miR-181a could remarkably reduce LPS-induced ALI in mice and A549 cells via targeting Bcl-2 [26]. PQ also caused the dysregulated expression of some miRNAs including miR-200a-3p, miR-21, miR-141-3p and miR-153, and only the level of miR-141-3p was influenced by EPCs. In addition, over-expression of miR-141-3p could abolish the effect of EPCs on the lung function. Hence, we confirmed that EPCs improved ALI via miR-141-3p.

In addition to miR-141-3p, the impact of PQ on Notch-Nrf2 axis was also reversed by EPCs. The Notch pathway modulates vital cellular biological processes such as proliferation and differentiation through communication between adjacent cells [27]. Notch signaling is reported to play a basic role in blood vessel development [28]. Notch signaling pathway is involved in the angiogenesis process of bone marrow-derived mesenchymal stem cell repairing smoke inhalation injury [29]. Nrf2 is ubiquitously expressed in cells and is hallmarked by a master modulator of the anti-oxidative response pathway, and Notch-Hesr-Nrf2 axis is potentially anti-oxidative pathway [30]. Notch signaling are Hes (Hairy and enhancer of split) and Hesr (Hes-related, also known as Hey/Herp/Hrt/Gridlock/Chf) family genes [31], and were inhibited that could reserve the reduced the effect of miR-141-3p knockdown on Nrf2 pathway in our study. Meanwhile, the inactivity of Notch signaling inhibited the same effect with miR-141-3p over-expression on inhibiting EPCs remission on PQ-induced ALI.

In summary, EPCs could provide therapeutic effect on PQ-induced ALI via miR-141-3p-Notch-Nrf2 Axis.
